# CodonShuffle: a tool for generating and analyzing synonymously mutated sequences

**DOI:** 10.1093/ve/vev012

**Published:** 2015-10-02

**Authors:** Daniel Macedo de Melo Jorge, Ryan E. Mills, Adam S. Lauring

**Affiliations:** ^1^Division of Infectious Diseases, Department of Internal Medicine, University of Michigan, Ann Arbor, MI 48109, USA,; ^2^Department of Computational Medicine and Bioinformatics, University of Michigan, Ann Arbor, MI 48109, USA,; ^3^Department of Human Genetics, University of Michigan, Ann Arbor, MI 48109, USA and; ^4^Department of Microbiology and Immunology, University of Michigan, Ann Arbor, MI 48109, USA

**Keywords:** synonymous mutation, RNA virus, synthetic, codon, bioinformatics

## Abstract

Because synonymous mutations do not change the amino acid sequence of a protein, they are generally considered to be selectively neutral. Empiric data suggest, however, that a significant fraction of viral mutational fitness effects may be attributable to synonymous mutation. Bias in synonymous codon usage in viruses may result from selection for translational efficiency, mutational bias, base pairing requirements in RNA structures, or even selection against specific dinucleotides by innate immune effectors. Experimental analyses of codon usage and genome evolution have been facilitated by advances in synthetic biology, which now make it feasible to generate viral genomes that contain large numbers of synonymous mutations. The generally pleiotropic effects of synonymous mutation on viral fitness have, at times, made it difficult to define the mechanistic basis for the observed attenuation of these heavily mutated viruses. We have addressed this problem by developing a bioinformatic tool for the generation and analysis of viral sequences with large-scale synonymous mutation. A variety of permutation strategies are applied to shuffle codons within an open reading frame. After measuring the dinucleotide frequency, codon usage, codon pair bias, and free energy of RNA folding for each permuted genome, we used *z*-score normalization and a least squares regression model to quantify their overall distance from the starting sequence. Using this approach, the user can easily identify a large number of synonymously mutated sequences with varying similarity to a wild-type genome across a range of nucleic-acid-based determinants of viral fitness. We believe that this tool will be useful in designing genomes for subsequent experimental studies of the fitness impacts of synonymous mutation.

## 1 Introduction

Issues of synonymous mutation and codon usage are fundamental to studies of molecular evolution. Synonymous mutation will not change the function of a protein, and in many cases, these mutations will be selectively neutral. This principle underlies commonly used metrics of positive and negative selection (L Hartl 1988; Yang 2006). However, many organisms and viruses exhibit biases in codon usage that are largely unexplained (Sharp et al. 1993; Jenkins and Holmes 2003; Belalov and Lukashev 2013). Often, codon bias can be ascribed to selection for translational efficiency, where highly expressed genes have codons that are well matched to the abundance of their respective tRNA in a given cell or tissue. The other main factor influencing base composition and codon usage is inherent mutational bias caused by sequence context or the polymerases themselves. Mutational pressure and selection for translational efficiency are not mutually exclusive, and many genomes have evidence of both processes (Plotkin and Kudla 2011).

Experiments in viral systems have suggested a number of additional reasons for codon usage bias within and between genomes. Much of this work has been performed in RNA viruses, where compact genomes, efficient natural selection, and a high level of host dependence have revealed situations where synonymous mutation can have a significant impact on fitness. The genomes of many RNA viruses fold into complex secondary and tertiary structures that are important for replication, translation, or evasion of host innate immunity (Simmonds and Smith 1999; Simmonds, Tuplin, and Evans 2004; Steil and Barton 2009). These structures often occur within open reading frames and may be perturbed by synonymous mutation. Codon usage may also be influenced by the dinucleotide frequency, and bias against CpG has been observed in a number of RNA viruses (Karlin, Doerfler, and Cardon 1994). While the reasons are not clear, it may reflect recognition of these sequences as a pathogen-associated molecular pattern by toll-like receptors (Rabadan, Levine, and Robins 2006; Burns et al. 2009; Wong et al. 2010). Similarly, avoidance of sequences targeted by host micro RNA may influence base and codon usage in RNA virus genomes. Sanjuan and coworkers have suggested that up to 18 per cent of the mutational fitness effects in RNA viruses may be due to selection at synonymous sites (Cuevas, Domingo-Calap, and Sanjuán 2012).

Experimental analyses of codon usage and genome evolution have been facilitated by advances in synthetic biology, which now make it feasible to generate viral genomes that contain large numbers of synonymous mutations (Wimmer and Paul 2011). This approach was initially applied to poliovirus, where shifting codon usage away from that of the natural human host reduced translational efficiency and virulence (Burns et al. 2006; Mueller et al. 2006). Alterations in codon or codon pair bias of poliovirus, vesicular stomatitis virus, influenza virus, and Dengue virus have all been used to rationally attenuate these agents for vaccine design (Nougairede et al. 2013; Nogales et al. 2014; Shen et al. 2015; Wang et al. 2015). In theory, these live, attenuated vaccines would have a low probability of reverting to virulence as each individual synonymous mutation has only a small impact on fitness. Subsequent work, however, has suggested that the process of fitness gain in these heavily mutated viruses is complex and may include compensatory mutations outside the synonymously mutated sequences (Bull, Molineux, and Wilke 2012; Nougairede et al. 2013).

Despite these advances, relatively little is known about the global fitness impact of large-scale synonymous mutation. One problem is that it is hard to alter one sequence determinant while keeping others intact. For example, shifting codon or codon pair bias may alter dinucleotide frequency, and it may be difficult to determine which distinct disruption leads to an observed fitness defect (Burns et al. 2009; Tulloch et al. 2014). We found that while codon usage was a determinant of viral mutational robustness, it was difficult to exclude pleiotropic effects of large-scale synonymous mutation on RNA structure or other sequence determinants (Lauring et al. 2012). Given the large size of synonymous sequence space, it should be possible to identify synonymously mutated sequences that differ from a wild type in only a single parameter, such as CpG frequency or codon pair bias.

Here, we describe a bioinformatic tool for the generation and analysis of viral sequences with large-scale synonymous mutation. We illustrate its features using the sequence coding for the poliovirus capsid, as poliovirus was the first virus to be chemically synthesized, and a number of synonymously mutated variants have been described (Cello, Paul, and Wimmer 2002; Burns et al. 2006, 2009; Mueller et al. 2006; Coleman et al. 2008). We employ four mutational strategies to generate thousands of permutations of an open reading frame (Belalov and Lukashev 2013). Each sequence contained hundreds of synonymous mutations. We incorporate a multifaceted approach to simultaneously evaluate the codon bias, codon pair bias, dinucleotide frequency, and free energy of RNA folding for these permuted sequences. By using *z*-score normalization of these metrics and a least-squares model for overall distance, users may identify sequences that are globally similar or dissimilar to the wild type. As this tool will also allow selection of sequences that are similar to wild type in all but one of the above metrics, we expect that it will facilitate experimental studies of the fitness impact of synonymous mutation.

## 2 Permutation of sequences

In many studies of synonymously mutated viruses, the goal has been to deoptimize the codon bias or codon pair bias of viral genomes leading to reduced translational efficiency (Burns et al. 2006, 2009; Mueller et al. 2006; Nogales et al. 2014; Shen et al. 2015; Wang et al. 2015). As we seek to identify sequences with preserved codon and codon pair bias, our permutation of the initial viral open reading frame relies on shuffling of existing codons. A number of permutation strategies are available that use distinct algorithms to generate sequences with large numbers of synonymous mutations. We chose four that are described in a recent study of viral codon usage (Belalov and Lukashev 2013).

**Figure 1.**
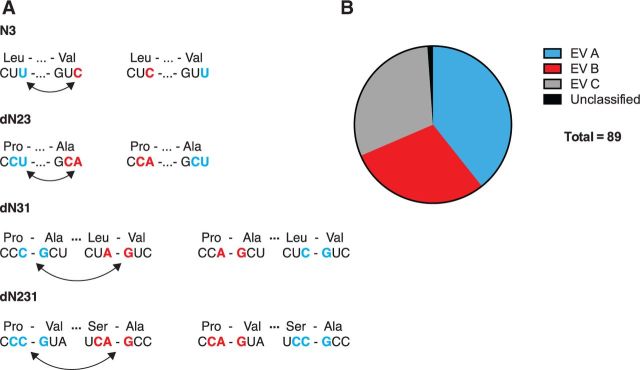
(A) Diagram of permutation approaches, adapted from Belalov and Lukashev (2013). (B) Subtype representation of eighty-nine enteroviral (EV) full-length capsid sequences used as reference set for analyses in Figs 2–6 below.

Because each approach generates synonymous mutations by shuffling the existing bases, the overall frequency of each is preserved (Belalov and Lukashev 2013). The N3 approach shuffles the third position of each codon throughout a sequence (Fig. 1A). The dN23 approach preserves the amino acid sequence while shuffling dinucleotides representing the second and third position of each codon. The dN31 approach shuffles dinucleotides corresponding to the third position of one codon and the first position of the next. The dN231 approach also permutes nucleotides within a codon pair, shuffling units consisting of the second and third position of the first codon and the first position of the next. Use of these different permutation strategies allowed us to generate a large number of candidate sequences that varied in various nucleic acid characteristics.

## 3 Analysis of permuted sequences

While permutation of a base sequence will preserve its overall nucleotide composition, shuffling of these bases has the potential to alter biologically relevant properties of the coding RNA. Among these are dinucleotide composition, codon bias, codon pair bias, and free energy of RNA folding. Therefore, the second step of our approach is to define how the permuted sequences differ from the wild-type input with respect to these metrics. We used the permutation strategies to create a large number codon-shuffled sequences based on the 2,643 base sequence that codes for the poliovirus capsid protein (Type I Mahoney strain, VP1–VP4). In our pilot study, we evaluated 1,000 sequences per permutation script.

We analyzed each group of 1,000 sequences for a variety of sequence-based metrics, plotting their distribution with respect to the wild type. To understand better the biological relevance of these differences, we also analyzed a set of enterovirus sequences downloaded from Genbank. Poliovirus is a Type C enterovirus, and the enterovirus genus includes a large number of closely related viruses with similar genome structures and replication strategies (Fields, Knipe, and Howley 2013). Out of 352 entries, we compiled a set of 89 full-length capsid sequences for comparison. Twenty-three of these were poliovirus with four Type I, four Type 2, and fifteen Type 3 sequences included (Fig. 1B).

### 3.1 Dinucleotide frequency

**Figure 2.**
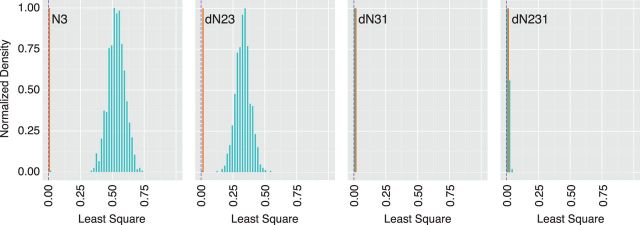
Dinucleotide bias of permuted sequences. Distribution of least squares values (*x* axis, see text) for 1,000 permuted sequences generated from each of the four permutation scripts, indicated in top left of each panel. Values for permuted sequences are shown in green and values for the reference set of eighty-nine enteroviruses are shown in orange. Purple dashed line is the value for the wild-type poliovirus capsid (Type 1, Mahoney). Of the four permutation approaches, dN31 and dN231 had little to no effect on the dinucleotide bias. This is consistent with a previously observed bias in GC content in the third codon and first codon positions of poliovirus (Belalov and Lukashev 2013).

An obvious and unavoidable consequence of nucleotide shuffling is alteration of the dinucleotide composition of a sequence. The frequency of a given dinucleotide can vary substantially in viral sequences, particularly across genera (Karlin, Doerfler, and Cardon 1994). It is well known that CpG and UpA are under-represented in many viruses. This bias may reflect the impact of these motifs on viral replication and the potential recognition of CpG, a pathogen-associated molecular pattern, by toll-like receptor 9. There are sixteen possible dinucleotides, and we measured bias in the usage of each dinucleotide in each candidate sequence by comparing the observed frequency of a given dinucleotide compared with its expected frequency given overall mononucleotide frequencies.

$${\text{Dinuc}}_{\text{AB}}=\;\frac{F_{\text{AB}}}{F_{\text{A}}\;x\;F_{\text{B}}},$$

where *F*_A_, *F*_B_, and *F*_AB_ are the frequencies of nucleotide A, nucleotide B, and the dinucleotide AB, respectively. Dinucleotide frequencies will also co-vary in a given sequence. Given a constant GC content, a bias toward CpG dinucleotides will lead to a bias away from GpC. Furthermore, inclusion of seventeen different measurements of dinucleotide bias in our final assessment of distance from wild type (see below) would overweight dinucleotide bias relative to other sequence-based determinants. We therefore used a least squares approach to compile the seventeen different measurements of dinucleotide bias into a single term.

$$\text{Dinucleotide bias}=\overset{16}{\underset{x=1}{\sum }}\sqrt{{\left(x_{\text{wt(AB)}}-x_{\text{Rd}(\text{AB)}}\right)}^{2}},$$

where *X*_wt(AB)_ and *X*_Rd(AB)_ are the frequency of a given dinucleotide (see above) in the wild-type and ‘random’ permuted sequence, respectively. The distributions of values for this composite metric of dinucleotide bias are shown in Fig. 2 for 1,000 sequences generated with each of the four permutation scripts. The overall bias of the wild type is shown for reference. The range in dinucleotide bias for our set of enterovirus capsid sequences is also shown.

### 3.2 Codon usage bias

Codon bias is a major determinant of translational efficiency, and optimization or deoptimization of codon usage is a common way to manipulate the replicative capacity, fitness, and virulence of a virus within a given host (Burns et al. 2006; Mueller et al. 2006; Plotkin and Kudla 2011). Because our goal was to generate sequences with replicative fitness similar to that of wild type, we incorporated two different measurements of codon bias into our algorithm. The simpler measurement, effective number of codons (ENC), reflects the number of the sixty-one non-termination codons that are used in a given sequence (Wright 1990).

$$\hat{N}c=2+\frac{9}{{\hat{F}}_{2}}+\frac{1}{{\hat{F}}_{3}}+\frac{5}{{\hat{F}}_{4}}+\frac{3}{{\hat{F}}_{6}},$$

where *F_i_* denotes the average homozygosity for the class with *i* synonymous codons, and the numerators in each term indicate the number of amino acids belonging to each class.

**Figure 3.**
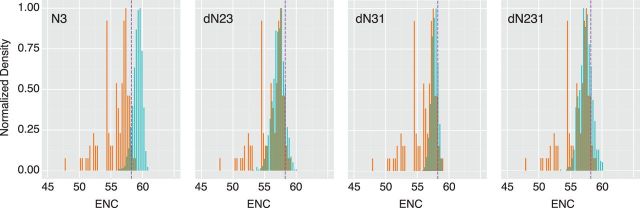
Codon bias of permuted sequences. Distribution of ENC values (*x* axis) for 1,000 permuted sequences generated from each of the four permutation scripts, indicated in top left of each panel. Values for permuted sequences are shown in green and values for the reference set of eighty-nine enteroviruses are shown in orange. Purple dashed line is the value for the wild-type poliovirus capsid (Type 1, Mahoney).

An ENC of twenty represents extreme bias as only one codon is used for each amino acid and a value of sixty-one suggests that there is no bias. The distributions in ENC values for our permuted sequences and the enterovirus reference set are shown in Fig. 3.

Because each of the four scripts is designed to predominantly shuffle codons, the overall codon bias is largely preserved.

The other commonly used metric of codon bias is the codon adaptive index (CAI), which measures the usage of codons in a given open reading frame relative to a reference set of highly expressed genes from a given organism (Sharp and Li 1987). In our case, we used a human reference set, as humans are the only known natural host for poliovirus.

$$w_{ij}=\;\frac{x_{ij}}{x_{i\text{max}}}$$

**Figure 4.**
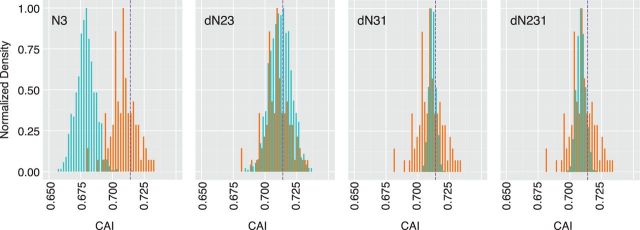
Codon bias of permuted sequences. Distribution of CAI values (*x* axis) for 1,000 permuted sequences generated from each of the four permutation scripts, indicated in top left of each panel. Values for permuted sequences are shown in green and values for the reference set of eighty-nine enteroviruses are shown in orange. Purple dashed line is the value for the wild-type poliovirus capsid (Type 1, Mahoney).

CAI=(∑k=1Llnwk)1L,

where *X_ij_* is the number of times that codon *i* for amino acid *j* occurs in the reference set of coding sequences, *L* is the number of codons in a gene, and *W_k_* is the weight of the *k*th codon in the gene sequence. The distributions in CAI values for our permuted sequences and the enterovirus reference set are shown in Fig. 4.

As for the ENC, these shuffling strategies largely preserve the codon bias of a sequence.

### 3.3 Codon pair bias

**Figure 5.**
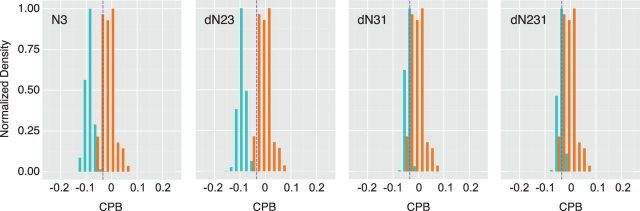
Codon pair bias of permuted sequences. Distribution of CPB values (*x* axis) for 1,000 permuted sequences generated from each of the four permutation scripts, indicated in top left of each panel. Values for permuted sequences are shown in green and values for the reference set of eighty-nine enteroviruses are shown in orange. Purple dashed line is the value for the wild-type poliovirus capsid (Type 1, Mahoney).

It has long been recognized that protein coding regions can exhibit bias in usage of synonymous codon pairs (Gutman and Hatfield 1989). While the biological relevance of this observed codon pair bias is unclear, some work suggests that it might influence translational efficiency by determining ribosomal A and P site occupancy (Coleman et al. 2008). As described previously, the first step in measuring codon pair bias is to generate a score for each codon pair. Like dinucleotide frequency, this codon pair score (CPS) compares the frequency of each codon pair relative to that expected by chance given the frequencies of each codon in a set of sequences.

$$\text{CPS}=\ln(\frac{F(\text{AB})}{\frac{F\left(\text{A}\right)\;x\;F(\text{B})}{F\left(\text{X}\right)\;x\;F(\text{Y})}\;x\;F(\text{XY})}),$$

where *F*(A), *F*(B), and *F*(AB) are the frequencies of codon A, codon B, and codon pair AB, respectively, and where *F*(X), *F*(Y), and *F*(XY) are the frequencies of amino acid A, amino acid B, and amino acid pair AB, respectively (Gutman and Hatfield 1989; Coleman et al. 2008). We used the CPS scores calculated from the human genome, as described in Coleman et al. (2008). The codon pair bias (CPB) for a given transcript is then the arithmetic mean of the CPS for each codon pair across the open reading frame.

$$\text{CPB}=\;\overset{k}{\underset{i=1}{\sum }}\frac{{\text{CPS}}_{i}}{k-1},$$

where *k* is the number of codon pairs in a given sequence. The distribution of CPB scores for our permuted sequences and the enterovirus reference set are shown in Fig. 5.

While both N3 and dN23 shuffling disrupted the codon pair bias of the poliovirus capsid, it was largely preserved when the dN31 and dN231 scripts were used to permute the sequences.

### 3.4 RNA folding

**Figure 6.**
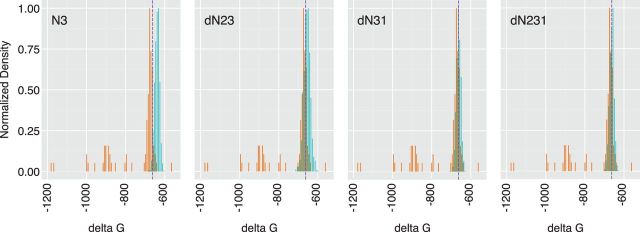
RNA structure in permuted sequences. Distribution of minimum free energy values (*x* axis) of 1,000 permuted sequences generated from each of the four permutation scripts, indicated in top left of each panel. Values for permuted sequences are shown in green and values for the reference set of eighty-nine enteroviruses are shown in orange. Purple dashed line is the value for the wild-type poliovirus capsid (Type 1, Mahoney).

Base pairing across an RNA sequence can result in complex secondary and tertiary RNA structures (Palmenberg et al. 2009). Because these structures often act as cis-acting regulatory sequences in RNA virus replication and translation, synonymous mutations can have profound effects on viral fitness. One advantage of the poliovirus system is that the capsid sequence is generally devoid of RNA secondary structure and tolerates large-scale synonymous mutation without apparent effects on replication (Burns et al. 2006; Mueller et al. 2006; Coleman et al. 2008; Lauring et al. 2012). Since this may represent a special case, we included an assessment of RNA folding free energy in our analysis algorithm. If permutation of the coding sequence either disrupts or creates a stable RNA structure, this would be identified as a change in free energy. We estimated the minimal free energy of folding using UNAFold (Markham and Zuker 2008), a more flexible and higher throughput software package based on the more commonly used Mfold algorithm (Zuker 2003). The folding free energies of our permuted sequences are shown in Fig. 6 relative to the wild type and enterovirus reference sets.

As above, the relatively narrow distribution of RNA folding free energy in the permuted sequences is consistent with the absence of stable structures across the poliovirus capsid sequence.

**Figure 7.**
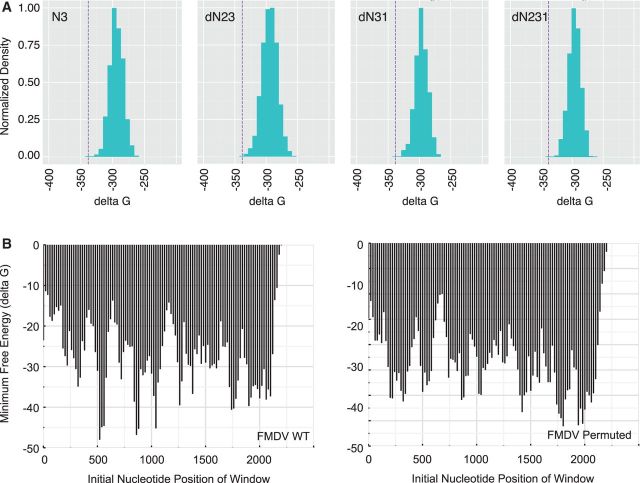
Minimum free energy of permuted FMDV sequences. (A) RNA structure in permuted sequences. Distribution of minimum free energy values (*x* axis) of 1,000 permuted sequences generated from each of the four permutation scripts, indicated in top left of each panel. Values for permuted sequences are shown in green. Purple dashed line is the value for the wild-type FMDV capsid sequence (Genbank KF152935.1). (B) Sliding window analysis, 100 nucleotides with eighty nucleotide overlap, of local RNA structure in the FMDV capsid sequence for the wild type (left), and one of the permuted sequences (right).

To demonstrate better this function of CodonShuffle, we performed an identical analysis of the corresponding capsid region of foot and mouth disease virus (FMDV), a virus with a high level of genome-scale-ordered RNA structure (Simmonds, Tuplin, and Evans 2004). Here, we found that permutation significantly altered the minimum free energy of RNA folding relative to the wild-type input sequence (Fig. 7A). All four permutation scripts had a similar effect, consistent with large-scale disruption of base pairing. While this global analysis of RNA structure is well suited to rapid analysis of large numbers of sequences, it is less sensitive for disruption of smaller but functionally important motifs. We therefore performed a sliding window analysis of a permuted FMDV sequence (Fig. 7B). Using a window size of 100 nucleotides and eighty nucleotide overlap as in (Coleman et al. 2008), we found significant perturbation of genome-scale-ordered RNA structure. This sliding window analysis can be performed within CodonShuffle on a limited subset of permuted sequences using ViennaRNA (Lorenz et al. 2011). There is currently no option to run it on larger datasets, as it is computationally costly and would require access to a large computer cluster.

## 4 Assessment of similarity to wild type

**Figure 8.**
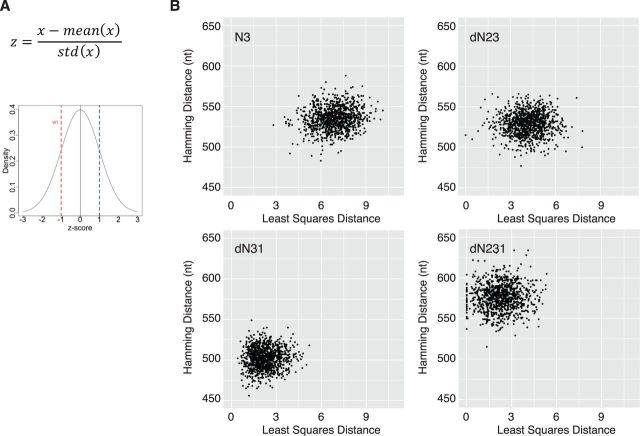
Assessment of overall similarity using a least squares model. (A) Calculation of *z*-score for each sequence in each distribution (blue dotted line) and the delta z relative to wild type (red dotted line), Figs 2–6. (B) Plots of Hamming distance versus Least Squares Distance, *D*, for 1,000 permuted sequences generated from each of the four permutation scripts, indicated in top left of each panel. The Hamming distance is in nucleotides across the 2,643 base capsid sequence of poliovirus.

As detailed above, the permuted sequences differ from the wild type across a range of metrics to varying degrees. In many cases, investigators would prefer to define a set of sequences that are similar to wild type across all of these genomic characteristics. Because each of the metrics has its own units and dynamic range, we used *z*-score normalization and a least squares model to combine them into a single measurement of distance relative to wild type. The distributions in Figs 2–6 were used to generate a *z*-score value (Fig. 8A) for each permuted sequence in a given distribution. A delta *z* for a given sequence and metric was then calculated as the difference in *z*-score value between the permuted sequence and the wild type. These delta *z* values were combined using least-square-based regression to generate a least squares distance (*D*).

$$\text{Least square dist }(D)=\sqrt{\begin{array}{l} {\left(Z\;{\mathrm{CPB}}_{\mathrm{wt}}\text{-}Z\;{\mathrm{CPB}}_{\mathrm{Rd}}\right)}^{2}\\ +{\left(Z\;{\mathrm{Mfold}}_{\mathrm{wt}}\text{-}Z\;{\mathrm{Mfold}}_{\mathrm{Rd}}\right)}^{2}\\ +{\left(Z\;{\mathrm{ENC}}_{\mathrm{wt}}\text{-}Z\;{\mathrm{ENC}}_{\mathrm{Rd}}\right)}^{2}\\ +{\left(Z\;{\mathrm{CAI}}_{\mathrm{wt}}-Z\;{\mathrm{CAI}}_{\mathrm{Rd}}\right)}^{2}\\ \begin{array}{l} +{\left(Z\;{\mathrm{Dinuc}}_{\mathrm{wt}}-Z\;{\mathrm{Dinuc}}_{\mathrm{Rd}}\right)}^{2} \end{array} \end{array}}$$

To define how close the permuted sequences were to wild type, we plotted *D* versus the Hamming nucleotide distance of each sequence (Fig. 8A).

While all four permutation approaches generated a large number of sequences that were quite different based on Hamming distance, they differed in how close the sequences were to wild type. We found that dN231 and d31 were the closest to wild type, followed by dN23 and N3. This is consistent with the manner in which each strategy permutes the input sequence and the effect of these permutations on the individual sequence determinants (Figs 2–6). For our purposes, dN231 was the best, as it generated sequences with the highest Hamming distance and lowest *D*.

## 5 Convergence on common solutions

**Figure 9.**
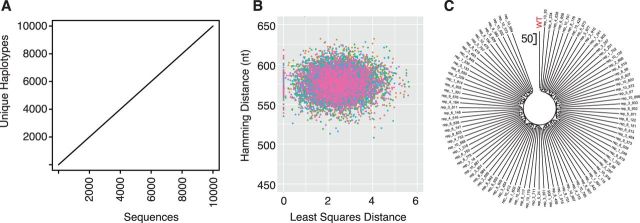
(A) Species (haplotype) accumulation curve with 10,000 sequences sampled (*x* axis) and number of unique haplotypes (*y* axis). Shown is the curve for sequences for the dN231 script. Curves for sequences generated with the three other scripts were identical. (B) Plots of Hamming distance versus least squares distance, *D*, for 10,000 permuted sequences generated from the dN231 script. Each sample of 1,000 sequences is shown in a different color. The Hamming distance is in nucleotides across the 2,643 base capsid sequence of poliovirus. (C) Neighbor-joining tree of ninety-eighty capsid sequences generated by dN231 with a *D* value of 0 (Figs 8 and 9B). Scale (no. nucleotides) is shown and the wild-type poliovirus sequence is indicated (red).

Synonymous sequence space for even a moderately sized open reading frame is quite large. Given that a peptide containing one of each of the twenty amino acids can be encoded by over 10^18^ distinct nucleic acid sequences, there will be many potential synonymous variants that code for the 881 amino acid poliovirus capsid. To determine whether our permutation approaches would converge on a set of common solutions, we ran them ten times, generating 10,000 total sequences with each strategy. Haplotype accumulation curves indicate that all the sequences in each of the four sets were unique (Fig. 9A).

A plot of Hamming distance versus overall distance (*D*) also demonstrates a range of optimal solutions for the dN231, which imposed the most constraints on permutation (Fig. 9B). Finally, we generated a neighbor joining tree of the ninety-eight nucleic acid sequences generated by dN231 that were closest to the wild type by our least squares distance metric (Fig. 9C). They were all distantly related to each other, with pairwise distances of 400–600. Therefore, CodonShuffle can quickly generate a large number of distinct synonymously mutated sequences that are similar to the wild type across a range of sequence determinants.

## 6 Installation and usage

We designed CodonShuffle to be run on a personal computer by users with limited experience in bioinformatics. It can be run on any Mac or Windows computer in a terminal window, and the entire dataset presented here can be obtained with just three commands. The user must have python installed and a number of additional programs. An ‘install dependencies’ script is provided to ensure these additional programs are installed and loaded in the appropriate directories. An ‘RNA sliding window’ script can be used to select individual sequences from the initial CodonShuffle output and to perform a sliding window analysis of their RNA structures (Fig. 7B). All other data are generated from the main ‘CodonShuffle’ script. Graphics are generated automatically, but users may also generate their own panels using the raw output data. Complete instructions for software download, installation, and usage are provided in an open-access github repository https://github.com/lauringlab/CodonShuffle

## Discussion

With recent advances in synthetic biology, it is now feasible to generate highly mutated viral genomes in a reasonable time frame (Wimmer and Paul 2011). This approach has been used to generate RNA viruses with large numbers synonymous mutations, often with the goal of predictable attenuation for vaccine design (Nogales et al. 2014; Shen et al. 2015; Wang et al. 2015). While most studies have sought to deoptimize codon and codon pair bias, synonymous mutation will often have pleiotropic effects on dinucleotide bias, RNA structure, and viral mutational robustness (Burns et al. 2009; Lauring et al. 2012; Tulloch et al. 2014). We have developed a flexible tool that will allow investigators to generate synonymously mutated sequences and to analyze them for differences in dinucleotide frequency, codon usage, codon pair bias, and free energy of RNA folding. A unique aspect of our algorithm is the *z*-score-based normalization of these diverse outputs and their ultimate combination into a single distance value derived by least squares regression. This distance value can be used to identify permuted sequences based on their overall similarity or dissimilarity to wild type.

We envision several ways in which this tool could be used to select individual sequences for synthesis and subsequent experimental analysis. In the first, one could choose sequences with a large Hamming distance and low *D* score. These sequences would be located in vastly different regions of sequence space yet share the same basic genomic characteristics of the wild type. Because synthetic viruses containing these permuted sequences would occupy distinct fitness landscapes, they could be used to study the impact of these landscapes on viral evolution (Lauring et al. 2012). For vaccine design, one might choose candidate sequences with a large Hamming distance and a high *D* score. Viruses based on these sequences would likely differ from wild type in many ways. This approach might lead to a greater level of attenuation than current examples, which have focused on codon bias, codon pair bias, or dinucleotide frequency alone. Some investigators may want to vary just one sequence determinant, dinucleotide frequency, for example, while holding all others constant. This could be accomplished by removing the dinucleotide frequency term from the final least squares regression and by using the N3 or dN23 permutation approaches only, which have the greatest impact on this particular determinant (Fig. 3). Finally, if perturbation of a single element, such as CpG content, is desired, the user could sort and interrogate the output .csv file, which provides all of the data on each permuted sequence. While CodonShuffle will run all permutation strategies and include all metrics by default, users can exclude either in the initial command.

The modular design of CodonShuffle also makes it a flexible tool. We included only measures of dinucleotide frequency, codon bias, codon pair bias, and folding free energy in our final calculation of distance. While these are perhaps the most studied parameters, synonymous mutation may also impact the tRNA adaptive index (Tuller et al. 2010), codon volatility (Plotkin and Dushoff 2003), or 5’–3’ codon bias of a sequence (Goodman, Church, and Kosuri 2013). These parameters could also be included in the least squares regression, provided a distribution of measurements can be obtained. In the case of localized codon volatility or 5’–3’ codon bias, one would need to perform a sliding window analysis to capture the variation in permuted sequences. A sliding window analysis could also be used to capture the effect of codon shuffling on local as opposed to global RNA structure (Coleman et al. 2008). Similarly, the modular design of CodonShuffle allows users to substitute their own tools. We used the commercial package, UNAfold, to analyze folding free energy given its facility with large numbers of sequences (Markham and Zuker 2008). Mfold and other freely available tools can be used to generate the requisite distributions for the *z*-score normalization and least squares regression (Zuker 2003). In the current version of CodonShuffle, users may select RNAfold package from Vienna RNA for this purpose (Lorenz et al. 2011).

We have implemented CodonShuffle in python and have its component scripts in a github repository that can be accessed anonymously, https://github.com/lauringlab/CodonShuffle. We believe that this tool will be useful in designing genomes for subsequent experimental studies of the fitness impacts of synonymous mutation.

## Data Availability

This software and associated data can be accessed anonymously at https://github.com/lauringlab/CodonShuffle *Conflict of interest*: None declared.
